# Supplementary material for: Molecular Linking Selectivity on Self-Assembled Metal-Semiconductor Nano-Hybrid Systems
Source: Nanomaterials (Basel). 2020 Jul 15;10(7):1378. doi: 10.3390/nano10071378 (PMC7407766; doi:10.3390/nano10071378)
Supplement: Supplementary file 1 [file nanomaterials-10-01378-s001.pdf]

Supporting Information

For

Molecular Linking Selectivity on Self-Assembled Metal-Semiconductor Nano-Hybrid Systems

Thinh Luong The Nguyen [1,†], Alba Gascón Nicolás [1,†], Tomas Edvinsson [2], Jie Meng [3], Kaibo Zheng [3,4], Mohamed Abdellah [1,5,6,*] and Jacinto Sá [1,6,7,*]

[1] Department of Chemistry—Ångström Laboratory, Uppsala University, Box 532, 751 20 Uppsala, Sweden; luongthethinh.nguyen.9036@student.uu.se (T.L.T.N.); albagasconnicolas@gmail.com (A.G.N.)
[2] Department of Materials Science and Engineering—Solid State Physics, Uppsala University, Box 35, 751 03 Uppsala, Sweden; tomas.edvinsson@angstrom.uu.se
[3] Department of Chemistry, Technical University of Denmark, DK-2800 Kongens Lyngby, Denmark; jmen@kemi.dtu.dk (J.M.); kaibo.zheng@chemphys.lu.se (K.Z.)
[4] Chemical Physics and NanoLund, Lund University, Box 124, 22100 Lund, Sweden
[5] Department of Chemistry, Qena Faculty of Science, South Valley University, 83523 Qena, Egypt
[6] Peafowl Solar Power AB, Henry Säldes väg 10, 756 43 Uppsala, Sweden
[7] Institute of Physical Chemistry, Polish Academy of Sciences, 01-224 Warsaw, Poland
* Correspondence: mo.abdellah@peafowlsolarpower.com (M.A.); jacinto.sa@kemi.uu.se (J.S.)
† Equal contribution.

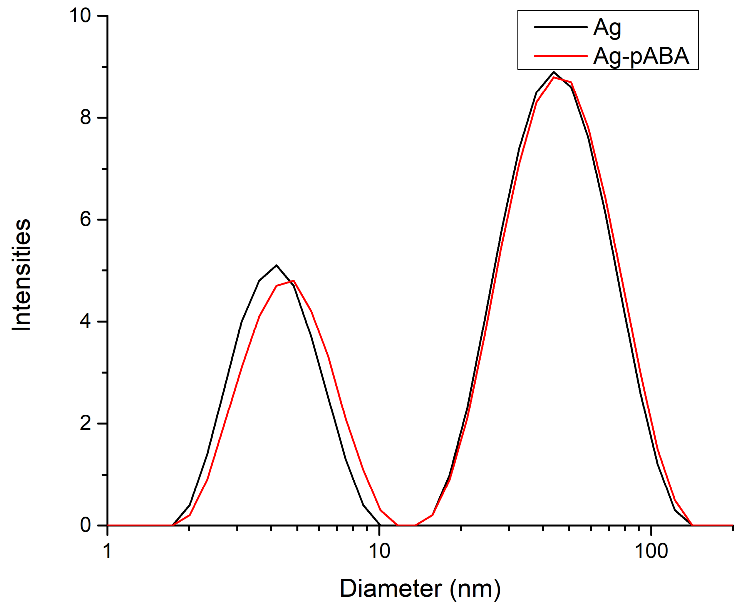

Figure S1. Dynamic light scattering (DLS) of Ag NPs (black trace), and Ag-pABA (red trace).

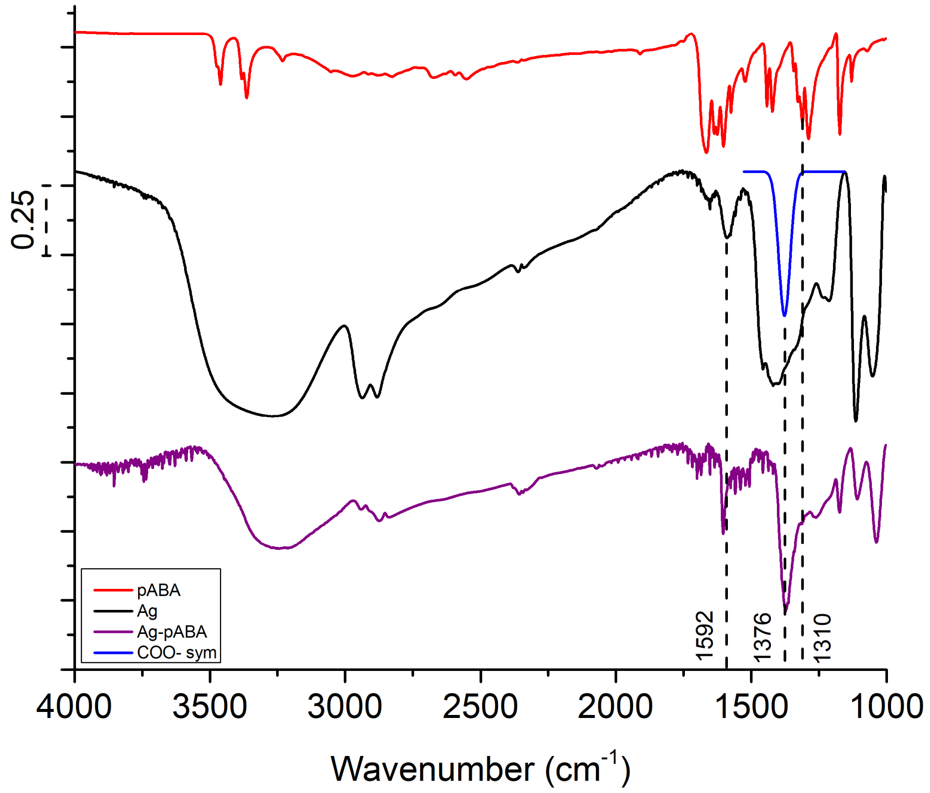

Figure S2. FTIR spectra of pABA (red trace), Ag NPs (black trace), and Ag-pABA (purple trace). The fit peak (blue trace) was obtained from deconvolution, showing COO- symmetric stretching.

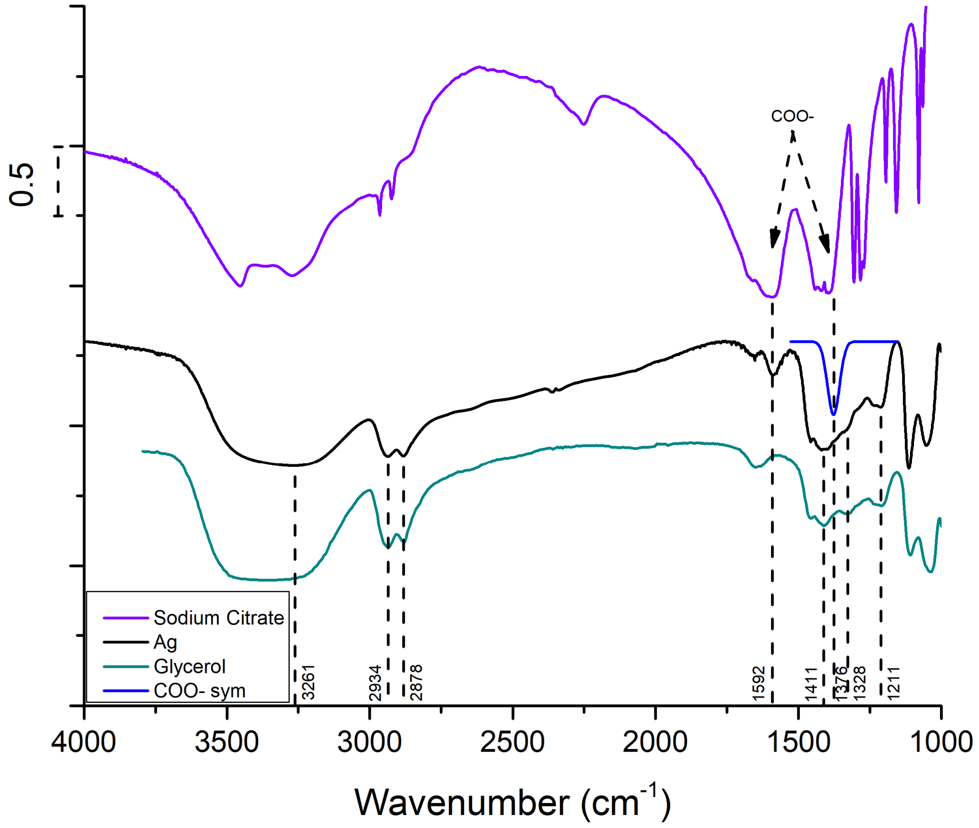

Figure S3. FTIR spectra of Ag NPs (black trace) along with sodium citrate (violet trace), and glycerol (cyan trace). Even after washing, the signal from glycerol is present in Ag NPs.

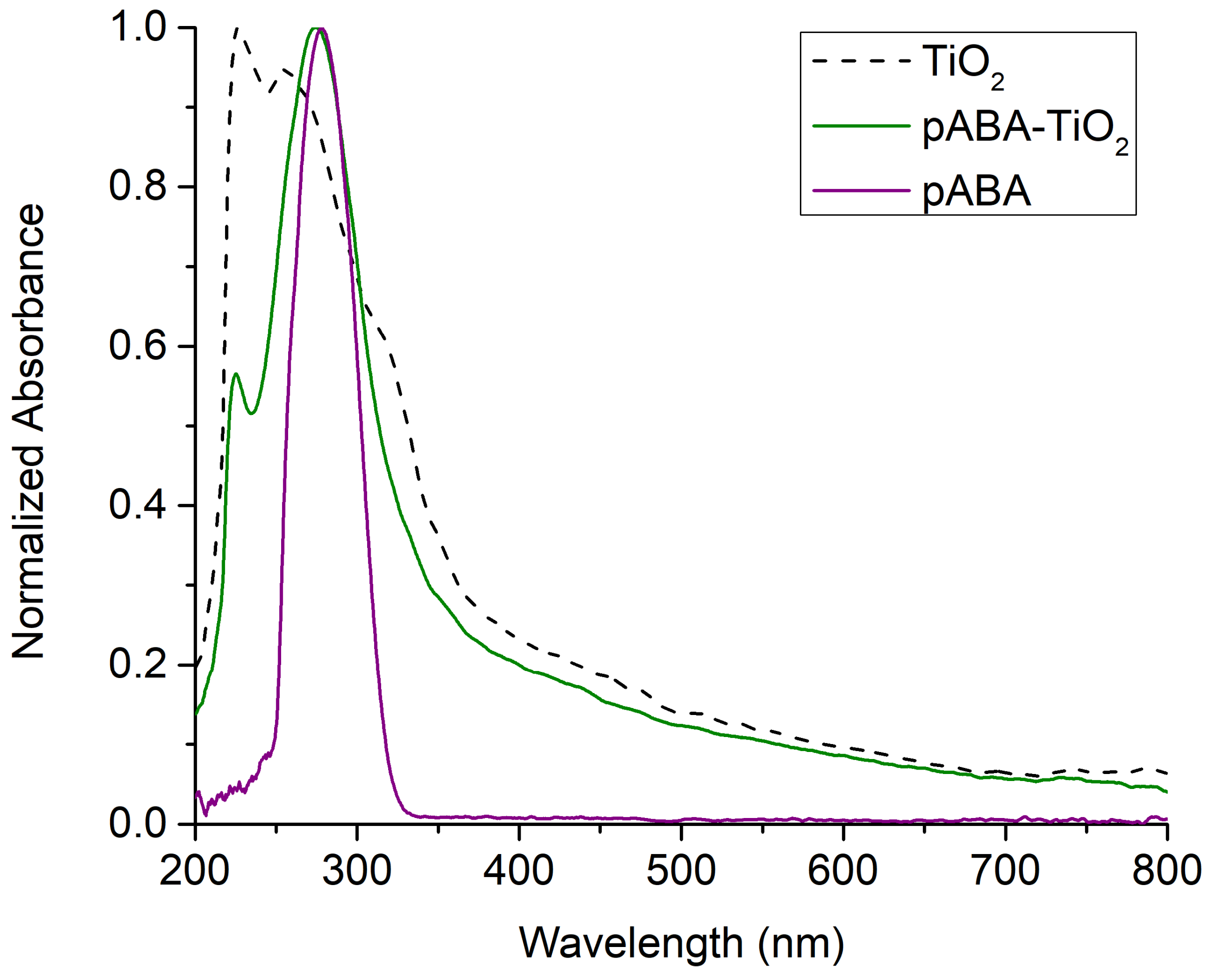

Figure S4. UV-Vis spectra of TiO_2 (black dashed trace), pABA (purple trace) and pABA on TiO_2 (green trace).

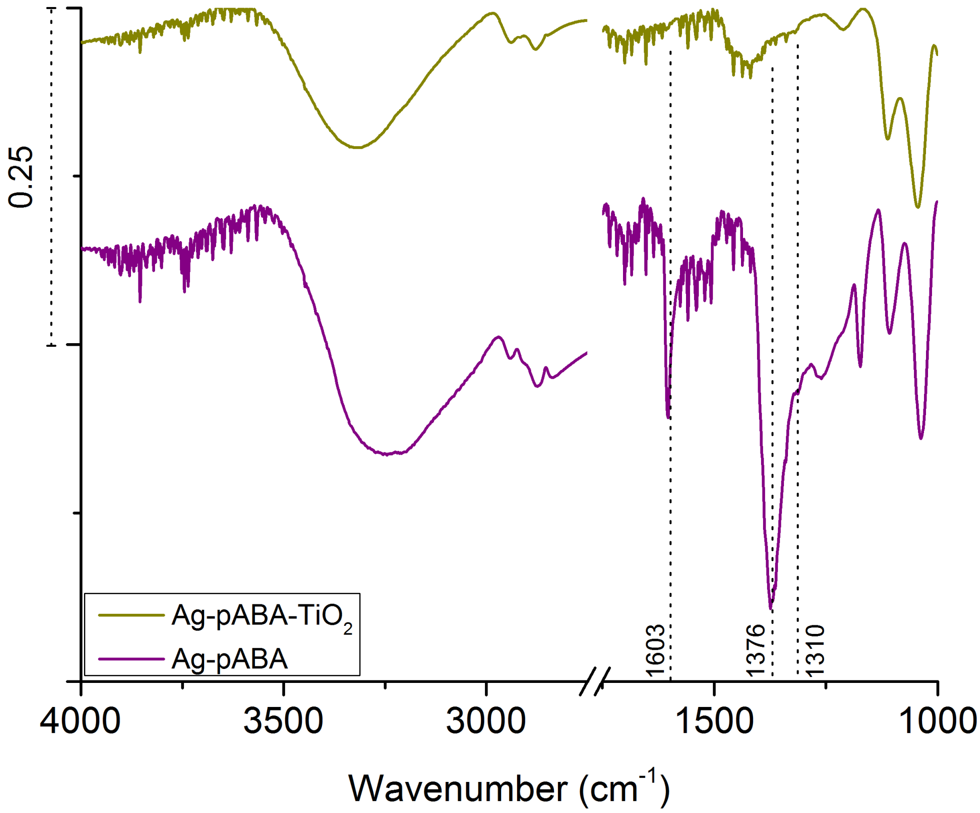

Figure S5. FTIR spectra of Ag-pABA-TiO_2 (dark yellow), and Ag-pABA (purple trace).

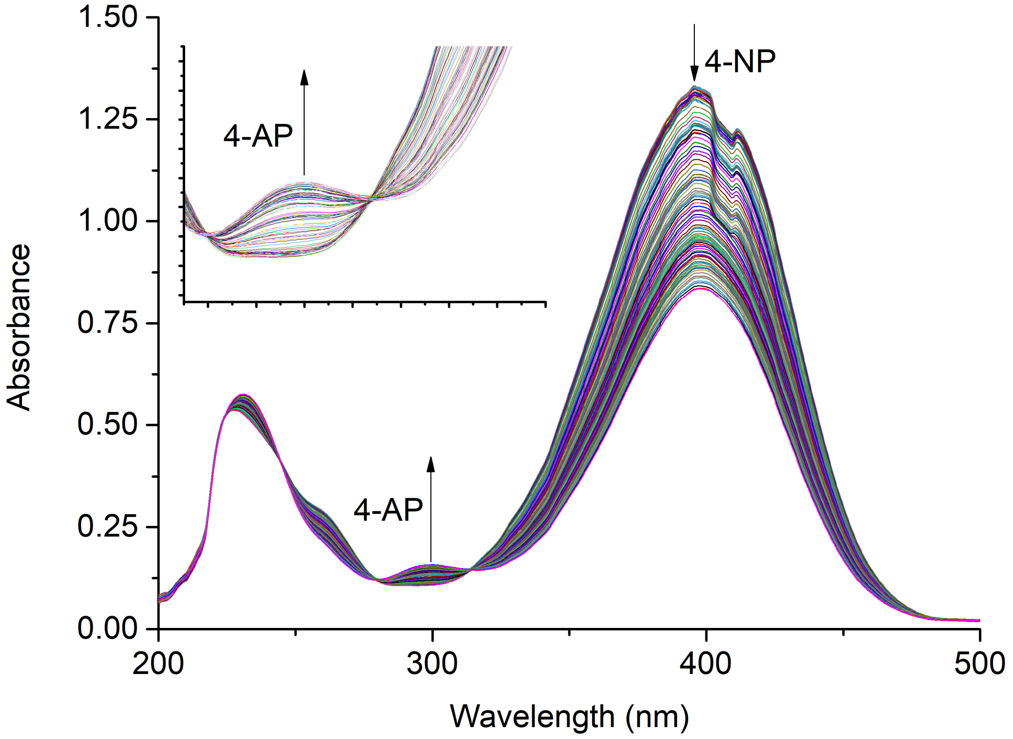

Figure S6. Temporal evolution of the UV-Vis spectra of the catalytic reaction using Ag-pABA-TiO_2 (10 μg/mL) and 405 nm laser excitation. Spectra recorded over 60 min.

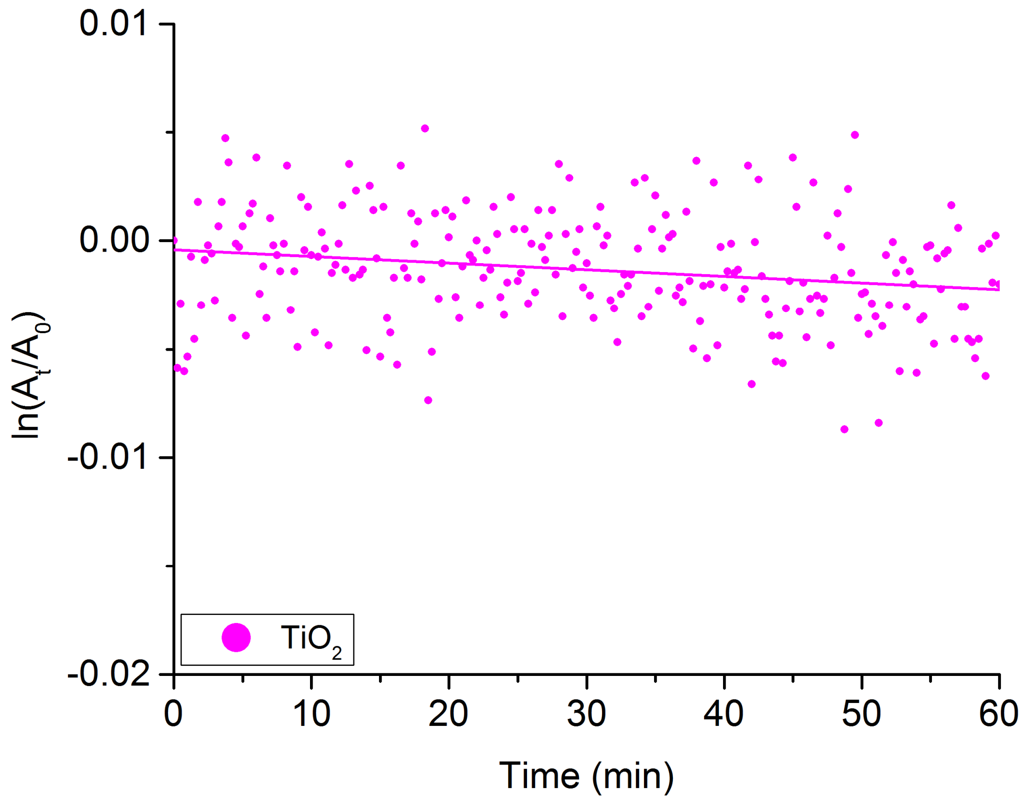

Figure S7. The disappearance rate of 4-NP using bare TiO_2, with laser excitation.
